# Allelopathic Effects of *Amomum villosum* Lour. Volatiles from Different Organs on Selected Plant Species and Soil Microbiota

**DOI:** 10.3390/plants11243550

**Published:** 2022-12-16

**Authors:** Xiang Zuo, Yanqian Wang, Hongyou Zhao, Guang Li, Yanfang Wang, Ge Li, Lixia Zhang, Weiwei Gao

**Affiliations:** 1 Institute of Medicinal Plant Development, Chinese Academy of Medical Sciences and Peking Union Medical College, Beijing 100193, China; 2 Yunnan Key Laboratory of Southern Medicine Utilization, Yunnan Branch of Institute of Medicinal Plant Development, Chinese Academy of Medical Sciences, Jinghong 666100, China

**Keywords:** allelopathy, *Amomum villosum*, GC–MS, volatile oils, volatile compounds, α-pinene, soil microorganisms

## Abstract

*Amomum villosum* Lour. is a perennial herb of the Zingiberaceae family, which is widely distributed in Xishuangbanna, Yunnan Province in Southwest China. Large amounts of volatile components contained in this plant enter the surrounding atmosphere and soil through volatilization, foliar leaching, root exudation, and residue decomposition. However, the ecological role of these compounds is currently unclear. The aim of this study was to compare the differences in the composition of volatile oils from stems, leaves, and young fruits of *A. villosum*, identify chemicals that had allelopathic effects, and explore the effects of the oil on the diversity and composition of soil microbiomes. Volatile oils were obtained by steam distillation and characterized by gas chromatography–mass spectrometry, and then were tested for allelopathic activity using seedlings of *Lactuca sativa* L. and *Lolium perenne* L. as test species. The results showed that the oils from stems and leaves were rich in monoterpene hydrocarbons, unlike the oxygenated monoterpenes which dominated oils from young fruits. Leaves > stems > young fruits: this was the order of the allelopathic effects of volatile oils from various *A. villosum* organs. Among the four main chemical components in the oils, only α-pinene, which is abundant in leaves, had a stronger allelopathic action than the crude oils, implying that it might be a potential allelochemical. Experiments on soil microorganisms indicated that 3.0 mg/mL oil had the greatest effect on the structure of the soil fungal community. It can be concluded that *A. villosum* is capable of releasing allelochemicals which affect the growth of other plant species and the diversity and community structure of soil microorganisms.

## 1. Introduction

Allelopathy is often defined as any direct or indirect, positive or negative effect by one plant species on another through the production and release of chemical compounds into the environment [1]. Plants release allelochemicals into the surrounding atmosphere and soil through volatilization, foliar leaching, root exudation, and residue decomposition [2]. As one group of important allelochemicals, volatile components enter the air and soil in similar ways: plants release volatiles into the atmosphere directly [3]; rainwater rinses these components (such as monoterpenes) out of the leaf secretory structures and surface waxes, providing the potential for volatile components into the soil [4]; plant roots could emit herbivore-induced and pathogen-induced volatiles into the soil [5]; these components in the plant litter are also released into the surrounding soil [6]. At present, volatile oils have been increasingly explored for their use in weed and pest management [7,8,9,10,11]. They are found to act by spreading in their gaseous state in the air and by transformation into other states into or onto the soil [3,12], playing an important role in inhibiting plant growth by interspecies interactions and altering the crop–weed plant community [13]. Several studies suggest that allelopathy may facilitate the establishment of dominance of plant species in natural ecosystems [14,15,16]. Therefore, dominant plant species can be targeted as potential sources of allelochemicals.

In recent years, allelopathic effects and allelochemicals have gradually received more and more attention from researchers for the purpose of identifying appropriate substitutes for synthetic herbicides [17,18,19,20]. In order to reduce agricultural losses, herbicides are increasingly used to control the growth of weeds. However, the indiscriminate application of synthetic herbicides has contributed to increased problems of weed resistance, the gradual degradation of the soil, and hazards to human health [21]. Natural allelopathic compounds from plants can offer considerable potential for the development of new herbicides, or as lead compounds toward identifying new, nature-derived herbicides [17,22].

*Amomum villosum* Lour. is a perennial herbaceous plant in the ginger family, growing to a height of 1.2–3.0 m in the shade of trees. It is widely distributed in South China, Thailand, Vietnam, Laos, Cambodia, and other Southeast Asian regions. The dry fruit of *A. villosum* is a kind of common spice because of its attractive flavor [23] and it represents a well-known traditional herbal medicine in China, which is widely used to treat gastrointestinal diseases. Several studies have reported that the volatile oils rich in *A. villosum* are the main medicinal components and aromatic ingredients [24,25,26,27]. Researchers found that essential oils of *A. villosum* exhibit contact toxicity against the insects *Tribolium castaneum* (Herbst) and *Lasioderma serricorne* (Fabricius), and strong fumigant toxicity against *T. castaneum* [28]. At the same time, *A. villosum* has a detrimental impact on the plant diversity, biomass, litterfall and soil nutrients of primary rainforests [29]. However, the ecological role of volatile oil and the allelopathic compounds are still unknown. In the light of previous studies into the chemical constituents of *A. villosum* essential oils [30,31,32], our objective is to investigate whether *A. villosum* releases compounds with allelopathic effects into the air and soil to help establish its dominance. Therefore, we plan to: (i) analyze and compare the chemical components of volatile oils from different organs of *A. villosum*; (ii) evaluate the allelopathy of volatile oils extracted and volatile compounds from *A. villosum*, and then identify the chemicals that had allelopathic effects on *Lactuca sativa* L. and *Lolium perenne* L.; and (iii) preliminarily explore the effects of oils from *A. villosum* on the diversity and community structure of microorganisms in the soil.

## 2. Results

### 2.1. Chemical Composition of the Volatile Oils from Different Plant Parts

The results of yield and chemical composition of the volatile oils from different plant parts are shown in Supplementary Table S1. The yield of oils from stems, leaves and young fruits was 0.15%, 0.40% and 0.50% (*v*/*w*, volume/fresh weight), respectively, which indicated that young fruits contain more volatiles than stems and leaves. The main constituents of stem oil were β-pinene (28.30%), β-phellandrene (24.65%), and α-pinene (16.26%), a composition which was similar to that of leaf oil: β-pinene (48.62%) and α-pinene (28.18%). The principal difference is that the main components of the volatile oils from young fruits were the oxygenated monoterpenes bornyl acetate (38.30%) and camphor (16.58%), whereas, in stem oil and leaf oil, monoterpene hydrocarbons were overwhelmingly dominant, accounting for 79.00% and 89.99%, respectively, while the second most abundant group was oxygenated monoterpenes, accounting for 8.53% (stems) and 4.02% (leaves). Unlike the oils in stems and leaves, the oil from young fruits was characterized by a high proportion of oxygenated monoterpenes (64.29%), followed by monoterpene hydrocarbons (32.55%), sesquiterpene hydrocarbons (1.18%) and oxygenated sesquiterpenes (0.57%). The chemical composition of the oil from young fruits showed the greatest diversity, with 54 compounds being identified.

### 2.2. Allelopathy of Volatile Compounds

**Figure 1:**
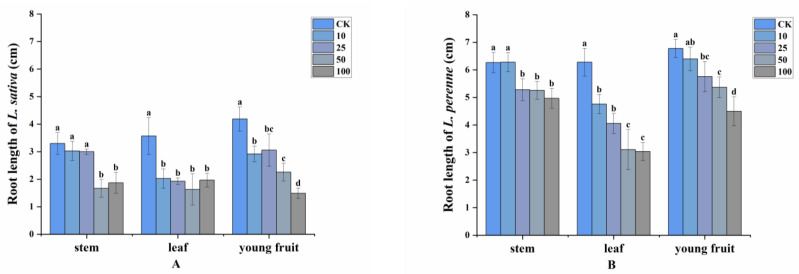
Allelopathic effects of *A. villosum* volatile compounds on the root length of (**A**): *L. sativa* and (**B**): *L. perenne* at 0 g/1.5 L (control, CK), 10 g/1.5 L, 25 g/1.5 L, 50 g/1.5 L and 100 g/1.5 L. Each value is the mean of three replicates ± SD (*n* = 30). Means with different lowercase letters indicate significant differences at *p* < 0.05 level according to ANOVA and Fisher’s LSD test.

The allelopathic effects on *L. sativa* and *L. perenne* of the volatile compounds released by different parts of *A. villosum* at 25 °C were explored. The root length of *L. sativa* was suppressed by 8.18%, 43.14%, or 30.31% when treated with 10 g fresh stems, leaves, or young fruits for each 1.5 L sealed container, respectively. As the weight of fresh plant material per container increased, the inhibitory effect on root length of volatile compounds from leaves on *L. sativa* barely changed. However, the root length was inhibited by 49.39% and 46.06% in the case of 50 g stems and young fruits for each 1.5 L container, respectively. When 100 g young fruits were added to the sealed box, 64.44% suppression of root length was achieved. In general, *L. perenne* was less sensitive than *L. sativa* in response to stems and young fruits, but not with respect to leaves. The root length of *L. perenne* decreased by only 20.73% and 33.63% at 100 g stems and young fruits per container, respectively. As the concentration increased, the root length inhibition rate by leaves increased from 24.2%, 35.35%, 50.48% to 51.59% (Figure 1).

**Figure 2:**
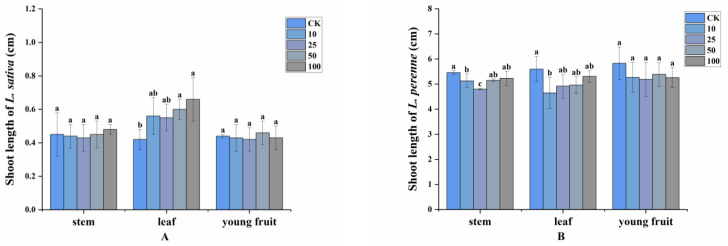
Allelopathic effects of *A. villosum* volatile compounds on the shoot length of (**A**): *L. sativa* and (**B**): *L. perenne* at 0 g/1.5 L (control, CK), 10 g/1.5 L, 25 g/1.5 L, 50 g/1.5 L and 100 g/1.5 L. Each value is the mean of three replicates ± SD (*n* = 30). Means with different lowercase letters indicate significant differences at the *p* < 0.05 level according to ANOVA and Fisher’s LSD test.

However, the effect of volatiles on shoot length was weaker than that on root length. For *L. sativa*, shoot length exhibited almost no difference in response to increasing concentration of stems and young fruits, except for a 57.14% increase in shoot length at 100 g leaves/1.5 L container. Unlike the promotion of shoot length for *L. sativa*, the shoot length of *L. perenne* was reduced by 12.09% at 25 g stems and 18.57% at 10 g leaves (Figure 2).

### 2.3. Allelopathic Effects of the Volatile Oils and Their Main Constituents

**Figure 3:**
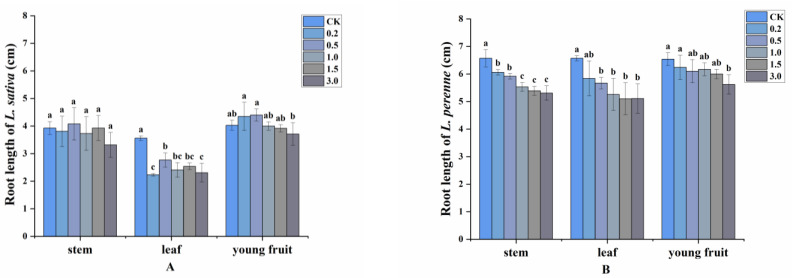
Allelopathic effects of volatile oils from *A. villosum* on the root length of (**A**): *L. sativa* and (**B**): *L. perenne*. Each value is the mean of three replicates ± SD (*n* = 30). Means with different lowercase letters indicate significant differences at *p* < 0.05 level according to ANOVA and Fisher’s LSD test.

Allelopathic activity of the volatile oils (concentrations tested ranged from 0.2 to 3.0 mg/mL), as well as the four major chemical components, were evaluated by comparing their effects on the root and shoot length of the two receiver plants (*L. sativa* and *L. perenne*). The inhibitory effect of volatile oils from stems and fruits on the root length of *L. sativa* was not significant, and the inhibition rate was only 15.52% and 7.94% for the two parts, respectively, at a concentration of 3.0 mg/mL, but the leaf oil markedly suppressed the root length by 37.36% at 0.2 mg/mL and 22.19%, respectively, at 0.5 mg/mL. On the whole, the inhibitory effects of oils on the root length of the monocot (*L. perenne*) were stronger than on those of the dicot (*L. sativa*). When the concentration of oils from stems, leaves and young fruits reached 3.0 mg/mL, the root length of *L. perenne* was significantly reduced by 19.18%, 22.37%, and 13.83%, respectively (Figure 3). In terms of the oils from different plant parts, leaf oil was more phytotoxic toward root growth than that from the other two plant organs (leaves > stems > young fruits). The shoot length of the test plants was also affected by the volatile oils but not significantly (the figure was omitted).

**Figure 4:**
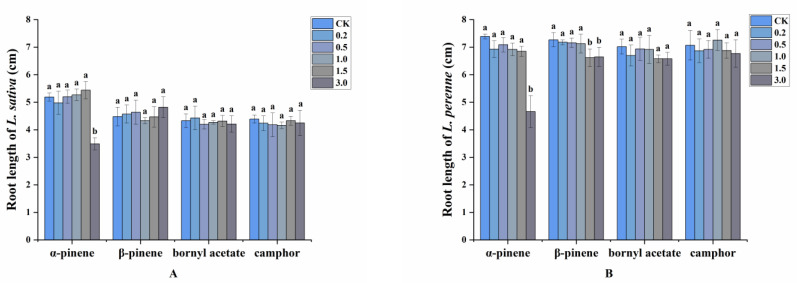
Allelopathic effects of four main volatile compounds on the root length of (**A**): *L. sativa* and (**B**): *L. perenne*. Each value is the mean of five replicates ± SD (*n* = 30). Means with different lowercase letters indicate significant differences at *p* < 0.05 level according to ANOVA and Fisher’s LSD test.

On the other hand, the major volatile compounds in the oils exhibited weaker allelopathic activity toward root growth than the volatile oils, apart from α-pinene. In the case of 3.0 mg/mL α-pinene, the root length of *L. sativa* was strikingly reduced by 32.76%. However, the other three compounds had almost no effect on root growth of *L. sativa*. When the concentrations reached 3.0 mg/mL, α-pinene and β-pinene significantly reduced root length by 36.94% and 8.53%, respectively, for *L. perenne*, whereas the effects of bornyl acetate and camphor were still not obvious. (Figure 4).

**Figure 5:**
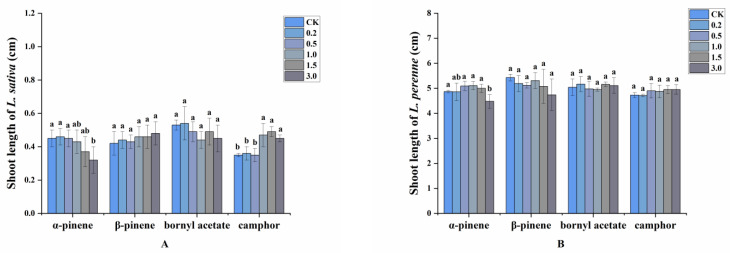
Allelopathic effects of the four main volatile compounds from the oil of *A. villosum* on the shoot length of (**A**): *L. sativa* and (**B**): *L. perenne*. Each value is the mean of five replicates ± SD (*n* = 30). Means with different lowercase letters indicate significant differences at *p* < 0.05 level according to ANOVA and Fisher’s LSD test.

Interestingly, the allelopathic effects on shoot length of the main volatile compounds were stronger than those of volatile oils from *A. villosum* from which they were isolated. When the concentration reached 1.0 mg/mL, α-pinene started to reduce the shoot length of *L. sativa* and the greatest inhibitory effect was achieved at 3.0 mg/mL, with 28.89% inhibition. On the contrary, camphor significantly increased shoot length of *L. sativa* by 40.00% at 1.5 mg/mL. Shoot length of *L. perenne* was prominently reduced by 8.01% at a high concentration (3.0 mg/mL) of α-pinene, and β-pinene at 3.0 mg/mL decreased the shoot length by 12.71%. Bornyl acetate and camphor had no significant allelopathic effects on this parameter (Figure 5).

### 2.4. Effects of Volatile Oil on Soil Microbiome

After data processing, we retained 1,324,268 sequences (bacteria) and 761,521 sequences (fungi) representing over 98.64% and 57.90% of the input sequences, respectively. The number of bacterial sequences varied from 29,739 to 38,196 per sample (mean = 33,956), whereas the number of fungal sequences varied from 13,170 to 31,094 per sample (mean = 19,526). These high-quality sequencing reads yielded a total of 35,653 (bacteria) and 2401 (fungi) amplicon sequence variants (ASVs). The data of ASVs is the basis for subsequent analysis.

Alpha-diversity, a comprehensive indicator of richness and evenness in species of community, refers to the diversity within a specific region or ecosystem [33]. Its community richness indexes mainly include Chao1 and ACE indexes [34,35], while the index of community diversity includes Shannon and Simpson indexes [36,37,38]. In this study, we measure the richness and diversity of soil microorganic communities through Chao1 and Shannon indexes, respectively. There were no significant differences between the groups added plant oil and the control group in the species number, Chao1 index, and Shannon index of soil bacteria (Table 1). However, on the 14th day of culture, compared to the control group, the number of fungi species increased significantly by 185% and the Chao1 index rose nearly twofold in the soil sample treated with 3.0 mg/mL volatile oil of *A. villosum*. These results indicate that the oils extracted from the *A. villosum* plant had a significant effect on the richness and diversity of soil fungi on the 14th day, whereas, the alpha-diversity of bacterial communities in the soil did not differ significantly between samples.

**Figure 6:**
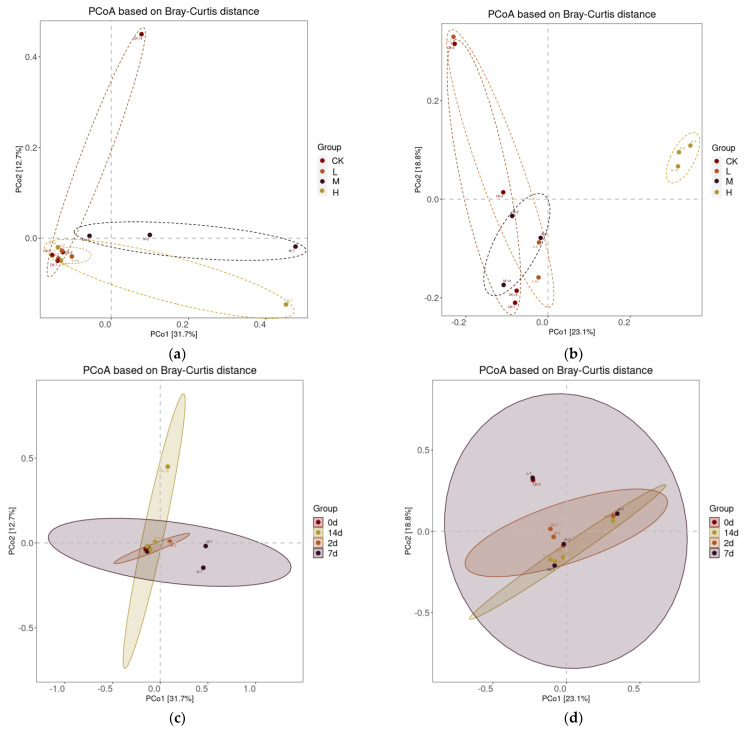
Principal Co−ordinates Analysis (PCoA) of bacteria (**a**,**c**) and fungi (**b**,**d**) based on Bray−Curtis distance. The picture (**a**,**b**) separately shows the difference of bacterial and fungal community composition of soil groups treated with different concentrations of volatile oil, while the picture (**c**,**d**) reveals the difference of bacterial and fungal community composition of soil groups at different treatment times, respectively. A point represents a sample, and points of the same color belong to a group. The solid circle represents the confidence ellipse with a 95% confidence interval, while the dotted circle is meaningless, just to better observe the same group of samples.

In a seminal paper, Whittaker [33] defined beta-diversity as “the extent of change in community composition or degree of community differentiation, in relation to a complex-gradient of environment or a pattern of environments”. The principal co-ordinates analysis (PCoA) is a common method to analyze beta-diversity [39], based on Bray-Curtis, Weighted Unifrac or Unweighted Unifrac distances. In the present study, beta-diversity is a comparative analysis of the microbial community composition of soil samples between different groups. PCoA plots (using Bray-Curtis distance) were constructed to visualize the separation between treatments. The closer the distance between samples, the more similar the community structure of soil microorganisms is indicated. From the principal co-ordinates analysis of soil samples under the treatment of different concentrations of the plant oil, we found that the group of high concentration had a great difference in fungal community compared with other treatment groups, which implied 3.0 mg/mL oil from *A. villosum* had a strong influence on the soil fungi. However, the soil samples with different treatment days did not exhibit visible disparity in the microbial community, which represented, within 14 days, the community structure of soil microorganisms was not related to treatment times (Figure 6).

**Figure 7:**
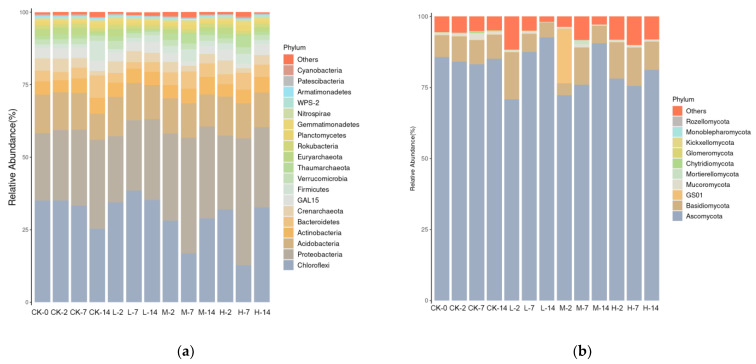
Relative abundance of the dominant bacteria (**a**) and fungi (**b**) at the phylum level.

**Figure 8:**
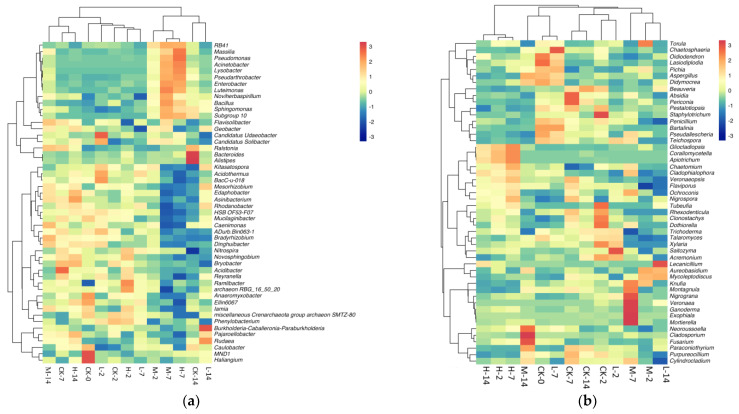
Heatmap of high−abundance bacteria (**a**) and fungi (**b**) at the genus level.

Microbial community composition was described by the mean relative abundance of sequences assigned to different taxa. By comparing the relative abundance of microorganisms in different soil samples, we could obtain a more comprehensive understanding of the effects of volatile oils from *A. villosum* on the community structure of soil microorganisms. In the control groups, the relative abundance of the phylum Chloroflexi decreased over time, while that of Proteobacteria increased. After seven days of culture, the relative abundance of the phylum Proteobacteria was 26.22% in the sample without the addition of oil, but in the M-7 (1.5 mg/mL oil) and H-7 (3.0 mg/mL oil) samples, their abundance reached 39.91% and 43.77%, respectively. The dominant fungi at phylum level were Ascomycota and Basidiomycota, with abundances differing in response to different oil treatments. On the second day of culture, the relative abundance of Ascomycota dropped in the group treated with volatile oil (Figure 7). The differences in the abundance of bacteria and fungi at the genus level in the 13 soil samples are illustrated in Figure 8. There was no prominent difference in the composition of bacterial community between soil samples. It was worth noting that the composition of fungal community in the soil treated with 3 mg/mL oil was significantly different from that of the no-oil group.

## 3. Discussion

The Zingiberaceae family has attracted increasing attention in allelopathic research because of the rich volatile oils and the aromaticity of its member species. Previous research had shown that the chemicals from *Curcuma zedoaria* (zedoary) [40], *Alpinia zerumbet* (Pers.) B.L.Burtt & R.M.Sm. [41] and *Zingiber officinale* Rosc. [42] of the ginger family have allelopathic effects on seed germination and seedling growth of maize, lettuce and tomato. Our current study is the first report on the allelopathic activity of volatiles from stems, leaves, and young fruits of *A. villosum* (a member of Zingiberaceae family). The oil yield of stems, leaves, and young fruits was 0.15%, 0.40%, and 0.50%, respectively, indicating that fruits produced a larger quantity of volatile oils than stems and leaves. The main components of volatile oils from stems were β-pinene, β-phellandrene and α-pinene, which was a pattern similar to that of the major chemicals of leaf oil, β-pinene and α-pinene (monoterpene hydrocarbons). On the other hand, the oil in young fruits was rich in bornyl acetate and camphor (oxygenated monoterpenes). The results were supported by the findings of Do N Dai [30,32] and Hui Ao [31] who had identified the oils from different organs of *A. villosum*.

There have been several reports on the plant growth inhibitory activities of these main compounds in other species. Shalinder Kaur found that α-pinene from eucalyptus prominently suppressed root length and shoot height of *Amaranthus viridis* L. at 1.0 μL concentration [43], and another study showed that α-pinene inhibited early root growth and caused oxidative damage in root tissue through increased generation of reactive oxygen species [44]. Some reports have argued that β-pinene inhibited germination and seedling growth of test weeds in a dose-dependent response manner by disrupting membrane integrity [45], altering the plant biochemistry and enhancing the activities of peroxidases and polyphenol oxidases [46]. β-Phellandrene exhibited maximum inhibition to the germination and growth of *Vigna unguiculata* (L.) Walp at a concentration of 600 ppm [47], whereas, at a concentration of 250 mg/m^3^, camphor suppressed the radicle and shoot growth of *Lepidium sativum* L. [48]. However, research reporting the allelopathic effect of bornyl acetate is scanty. In our study, the allelopathic effects of β-pinene, bornyl acetate and camphor on root length was weaker than for the volatile oils except for α-pinene, whereas leaf oil, rich in α-pinene, was also more phytotoxic than the corresponding volatile oils from the stems and fruits of *A. villosum*, both findings indicating that α-pinene might the important chemical for allelopathy by this species. At the same time, the results also implied that some compounds in the fruit oil that were not abundant might contribute to the production of the phytotoxic effect, a finding which needs further research in the future.

Under normal conditions, the allelopathic effect of allelochemicals is species-specific. Jiang et al. found that essential oil produced by *Artemisia sieversiana* exerted a more potent effect on *Amaranthus retroflexus* L. than on *Medicago sativa* L., *Poa annua* L., and *Pennisetum alopecuroides* (L.) Spreng. [49]. In another study, the volatile oil of *Lavandula angustifolia* Mill. produced different degrees of phytotoxic effects on different plant species. *Lolium multiflorum* Lam. was the most sensitive acceptor species, hypocotyl and radicle growth being inhibited by 87.8% and 76.7%, respectively, at a dose of 1 μL/mL oils, but hypocotyl growth of cucumber seedlings was barely affected [20]. Our results also showed that there was a difference in sensitivity to *A. villosum* volatiles between *L. sativa* and *L. perenne*.

The volatile compounds and essential oils of the same species can vary quantitatively and/or qualitatively because of growth conditions, plant parts and detection methods. For example, a report demonstrated that pyranoid (10.3%) and β-caryophyllene (6.6%) were the major compounds of the volatiles emitted from leaves of *Sambucus nigra*, whereas benzaldehyde (17.8%), α-bulnesene (16.6%) and tetracosane (11.5%) were abundant in the oils extracted from leaves [50]. In our study, volatile compounds released by the fresh plant materials had stronger allelopathic effects on the test plants than the extracted volatile oils, the differences in response being closely related to the differences in the allelochemicals present in the two preparations. The exact differences between volatile compounds and oils need to be further investigated in subsequent experiments.

Differences in microbial diversity and microbial community structure in soil samples to which volatile oils had been added were related to competition among microorganisms as well as to any toxic effects and the duration of volatile oils in the soil. Vokou and Liotiri [51] found that the respective application of four essential oils (0.1 mL) to cultivated soil (150 g) activated respiration of the soil samples, even the oils differed in their chemical composition, suggesting that plant oils are used as a carbon and energy source by occurring soil microorganisms. Data obtained from the current study confirmed that the oils from the whole plant of *A. villosum* contributed to the obvious increase in the number of the soil fungal species by the 14th day after oil addition, indicating that the oil may provide the carbon source for more soil fungi. Another study reported a finding: soil microorganisms recovered their initial function and biomass after a temporary period of variation induced by the addition of *Thymbra capitata* L. (Cav) oil, but the oil at the highest dose (0.93 µL oil per gram of soil) did not allow soil microorganisms to recover the initial functionality [52]. In the current study, based on the microbiological analysis of the soil after being treated with different days and concentrations, we speculated that the soil bacterial community would recover after more days. In contrast, the fungal microbiota cannot return to its original state. The following results confirm this hypothesis: the distinct effect of high-concentration of the oil on the composition of soil fungal microbiome was revealed by principal co-ordinates analysis (PCoA), and the heatmap presentations confirmed again that the fungal community composition of the soil treated with 3.0 mg/mL oil (namely 0.375 mg oil per gram of soil) at the genus level differed considerably from the other treatments. Presently, the research about the effects of the addition of monoterpene hydrocarbons or oxygenated monoterpenes on soil microbial diversity and community structure is still scarce. A few studies reported that α-pinene increased soil microbial activity and relative abundance of Methylophilaceae (a group of methylotrophs, Proteobacteria) under low moisture content, playing an important role as a carbon source in drier soils [53]. Similarly, volatile oil of *A. villosum* whole plant, containing 15.03% α-pinene (Supplementary Table S1), obviously increased the relative abundance of Proteobacteria at 1.5 mg/mL and 3.0 mg/mL, which suggested that α-pinene possibly act as one of the carbon sources for soil microorganisms.

The volatile compounds produced by different organs of *A. villosum* had various degrees of allelopathic effects on *L. sativa* and *L. perenne*, which was closely related to the chemical constituents that *A. villosum* plant parts contained. Although the chemical composition of the volatile oil was confirmed, the volatile compounds released by *A. villosum* at room temperature are unknown, which need the further investigation. Moreover, the synergistic effect between different allelochemicals is also worthy of consideration. In terms of soil microorganisms, to explore the effect of the volatile oil on soil microorganisms comprehensively, we still need to conduct more in-depth research: extend the treatment time of volatile oil and discern variations in chemical composition of the volatile oil in the soil on different days.

## 4. Materials and Methods

### 4.1. Plant Materials

Stems and leaves of *A. villosum* were collected in Xishuangbanna South Medicine Garden located in Yunnan Province of China, and young fruits of *A. villosum* were collected from the Mengyang planting base in Jinghong City in Xishuangbanna, in July, 2022. The collected plant was identified by Professor Wang from the Yunnan Branch of the Institute of Medicinal Plant Development. Seeds of *L. sativa* and *L. perenne* were obtained from a seed company in Jiangsu Province. The two species were chosen for our growth experiments because of their uniform seedling emergence and high germination rates. For these reasons, they are frequently used as standard monocotyledonous and dicotyledonous plants, respectively, in allelopathic research [54,55,56,57].

### 4.2. Isolation of the Volatile Oils

Fresh stems, leaves and whole plants were used to extract volatile oils after being crushed by a pulverizer, but young fruits only needed to be cut into small pieces before oil extraction due to their high–water content. Then, 200 g of fresh stems, leaves, young fruits or whole plants (including rhizomes, stems and leaves) were each mixed with 600 mL water and heated for 5 h, according to the method of steam distillation described in the Chinese Pharmacopoeia [58]. The oil obtained from each tissue sample was dried over anhydrous sodium sulfate anhydrous and preserved at 4 °C for further use. The procedures were repeated three times.

### 4.3. Gas Chromatography–Mass Spectrometry Analysis

Gas chromatography–mass spectrometry analysis of the essential oils from the four tissue samples was carried out using a 5975C Agilent mass spectrometer (Agilent, Palo Alto, CA, USA) and a 7890A Agilent gas chromatograph apparatus (Agilent, Palo Alto, CA, USA) equipped with an Agilent HP-5MS (30 m × 250 µm × 0.25 µm) capillary column (Agilent, Palo Alto, CA, USA). The column temperature program was 60 °C increasing at a rate of 7 °C/min up to 200 °C, and then at a rate of 10 °C/min up to 260 °C, which was maintained for 2 min. The carrier gas was helium at a 1 mL/min flow rate. Split-mode injection (ratio 1:30) was employed. Mass spectra were collected over the range 50–500 *m/z* with an ionizing voltage of 70 eV. Samples were diluted in hexane before injection. The obtained individual compounds were identified by comparing their retention time (relative to a homologous series of n-alkanes) and mass spectrum with the NIST 08 (National Institute of Standards and Technology) library. The peak areas were used to calculate the relative percentages of the volatile oil constituents.

### 4.4. Allelopathy of Volatile Compounds

Fresh plant parts (stems, leaves and young fruits) of *A. villosum* were cut up and then put into airtight plastic containers (18 cm × 13 cm × 6.5 cm, volume 1.5 L) at the following concentrations (0 g, 10 g, 25 g, 50 g and 100 g per container) according to published literature [59,60]. After cleaning and surface-sterilization, seeds of *L. sativa* and *L. perenne* were separately placed in petri dishes at 25 ± 2 °C to promote germination. Then, 30 germinated, similar-sized seedlings of the test species (germinal length 1–2 mm), were selected and put into uncovered petri dishes (12 cm in diameter) with filter paper. Each petri dish received 5 mL ultrapure water and was then transferred into the plastic containers, one dish per container). The containers were placed in an illuminated, temperature-controlled incubator at 25 ± 2 °C with a light/dark photoperiod of 12:12, and were opened for 15 min each day to provide fresh air for the seedlings. Seedlings (root length, shoot length) were measured after 5 days (*L. sativa*) or 6 days (*L. perenne*) incubation. The experiment was repeated three times.

### 4.5. Allelopathic Effects of the Volatile Oils and Main Compounds

The allelopathic effects of the three oils (leaf, stem, fruit) as well as their major compounds (α-pinene, β-pinene, bornyl acetate and camphor) were estimated by conducting bioassays against a typical dicot (*Lactuca sativa*) and monocot plant species (*Lolium perenne*). The essential oils and their main compounds were dissolved in 0.5% acetone and then diluted in ultrapure water to produce 0.2, 0.5, 1.0, 1.5, and 3 mg/mL solutions, while a control treatment was achieved by ultrapure water. Previous experiments showed that acetone at 0.5% concentration had no significant effect on the seedling growth of testing plants [47]. As described earlier, 30 homogeneous germinated seedlings were put into petri dishes. Subsequently, 5 mL of each concentration was added to the culture dishes (containing filter paper). The dishes were sealed with plastic wrap and incubated in the growth chamber under the temperature and light conditions of SubSection 4.4. Relevant measurements of the seedlings were carried out after 5 days (*L. sativa*) or 6 days (*L. perenne*). Three (volatile oil) and five (main compounds) replicates were conducted for allelopathic bioassays, respectively.

### 4.6. Effects of the Volatile Oil on Soil Microorganisms

Soil samples were collected from an open space (which had never been treated with herbicides) in the South Medicine Garden in Xishuangbanna, China (22°0′13″ N, 100°47′17″ E). After randomly selecting three sample sites, we took the soil from the horizon at a depth of 3 to 8 cm as samples. Three soil samples were mixed and passed through a 2 mm mesh sieve. A subsample (1.5 g) of soil was taken as the control group and labeled CK-0, then stored at −80 °C for subsequent experiments. The remaining soil was evenly divided into four parts (numbered CK, L, M and H) and respectively placed in the culture bottles (40 g soil per bottle). The volatile oil of whole plants (including rhizomes, stems and leaves) of *A. villosum* was prepared for solutions at concentrations of 0.5, 1.5 or 3.0 mg/mL. Subsequently, 5 mL sterile water and three concentrations of oils from the whole plants were separately added to the four bottles. After mixing the oil solutions thoroughly with soils, we put them into an incubator at 25 ± 2 °C (light/dark = 14:10). Soil samples of 1.5 g were removed from each culture bottles on the 2nd, 7th, and 14th days, and then stored at −80 °C. Each soil sample (1.5 g) was equally divided into 3 parts as three replicates, respectively. A total of 13 soil samples was obtained for DNA extraction and high-throughput sequencing analysis of microorganisms.

The soil samples were sent to a commercial laboratory (Rhonin Biosciences, Chengdu, China) for sequencing analysis of the soil microorganisms. The DNA-extraction was performed using a Zymo Research BIOMICS DNA Microprep Kit (Cat#D4301) (Zymo Research, Los Angeles, CA, USA), according to the manufacturer’s protocols. The purity and integrity of the extracted DNAs were checked using 0.8% agarose gel electrophoresis. After assessing the concentration of each DNA samples, we stored them at −20 °C for further PCR amplification. The bacterial universal V4 region of the 16S rRNA gene was amplified with the PCR primers 515F (5′-GTGYCAGCMGCCGCGGTAA-3′) and 806R (5′-GGACTACHVGGGTWTCTAAT-3′) as previously described [61,62], while the fungal universal ITS2 region was amplified with the PCR primers ITS3 (5′-GATGAAGAACGYAGYRAA-3′) and ITS4(5′-TCCTCCGCTTATTGATATGC-3′) [63]. The individual PCR reactions were performed in 50 µL final volume and contained: 5 µL of 10× PCR Buffer for KOD-Plus-Neo, 5 µL of a 2 mM dNTPs solution, 3 µL of a 25 mM MgSO_4_ solution, 1.5 µL of 10 µM solutions of the PCR primers, 1 µL of KOD-Plus-Neo polymerase and 2 µL of DNA templates. Reactions were performed in an Applied Biosystems PCR System 9700 (Applied Biosystems, New York, NY, USA) using the following programme: 94 °C (1 min), followed by 30 cycles of 94 °C (20 s), 54 °C (20 s) and 72 °C (30 s), and a final step of 72 °C (5 min). To minimize potential biases originating during PCR amplifications, individual reactions were performed in triplicate. Three PCR products per sample were pooled, detected, purified and quantified by real-time quantified PCR. Once quantified, the PCR product from each group was pooled to a new tube in an equimolar ratio to generate amplicons libraries. Samples were sequenced on the HiSeq PE250 instrument (Illumina, San Diego, CA, USA) using a 2 × 250 nucleotide paired reads chemistry.

### 4.7. Statistical Analysis

The bioassay experiments followed a randomized design with three replications and 30 seedlings for each replicate. Results were expressed as mean ± standard deviation of the mean and analyzed by one-way ANOVA (*p* < 0.05) and Fisher’s Least Significant Difference (LSD) test at *p* < 0.05 level. All the statistical analyses were performed with SPSS version 26.0 (IBM, Armonk, NY, USA).

The processing of the raw sequences obtained through high-throughput sequencing was performed using the Quantitative Insights into Microbial Ecology (QIIME 2020.2) [64]. Reads with average quality lower than 30, length less than 200 bp and ambiguous bases were discarded before clustering. After completing the species annotation of feature sequences and the construction of ASV table and evolutionary tree, we performed alpha-diversity, beta-diversity, and community composition analysis on the obtained data by R 4.0.5 version. The result of alpha-diversity was expressed as mean ± standard deviation of the mean and analyzed by Dunn’s post hoc test at *p* < 0.05 level. Chao1 and Shannon indexes were calculated by using the vegan package. PCoA plots (based on Bray-Curtis distance using the vegdits function in vegan) were constructed in order to visualize the separation between different groups.

## 5. Conclusions

Our results showed that *A. villosum* was able to synthesize and release volatiles with allelopathic potential into the surrounding environments, which then affected the growth of other plants; this is a possible strategy by which *A. villosum* can promote its own establishment as a dominant species. Comparing the allelopathic effects of oils from different parts, we found that: leaves > stems > young fruits. Only α-pinene, one of the main constituents of oils from *A. villosum*, had stronger allelopathic suppression than oils, which implied that α-pinene might be the most potent allelochemical. Stronger allelopathic effects of volatile compounds than oils suggested that volatile compounds released at 25 °C might play a more important role in the growth inhibition of *L. sativa* and *L. perenne* seedlings. Experiments on soil microorganisms indicated that the effect of different concentrations of volatile oil from *A. villosum* on the soil microorganisms was different, but within 14 days, the impact of various treatment intervals was not obvious. Our conclusion is that some volatile compounds of *A. villosum* have allelopathic potential to the growth of other plant species and soil microbiota.

## Figures and Tables

**Table 1:** Alpha-diversity indexes of soil microorganisms in soil samples exposed to oil from *A. villosum* whole plant.

Samples	Oil (mg/mL)	Days	Observed Species		Chao1 Index		Shannon Index	
			Bacteria	Fungi	Bacteria	Fungi	Bacteria	Fungi
CK-0	-	0	1461 ± 211 ab	135 ± 42 bc	1513.94 ± 219.21 ab	149.72 ± 53.23 bcd	5.63 ± 0.43 a	3.45 ± 0.14 ab
CK-2	0	2	1313 ± 89 ab	127 ± 30 bc	1349.57 ± 87.86 ab	146.13 ± 47.40 bcd	5.49 ± 0.42 a	2.83 ± 0.35 abc
L-2	0.5	2	1472 ± 229 ab	111 ± 23 bc	1527.52 ± 235.31 ab	127.54 ± 31.80 bcd	5.37 ± 0.83 a	3.16 ± 0.22 abc
M-2	1.5	2	1263 ± 144 ab	73 ± 8 c	1295.95 ± 151.30 ab	78.72 ± 13.43 d	5.70 ± 0.08 a	2.46 ± 0.42 abc
H-2	3.0	2	1359 ± 230 ab	153 ± 43 b	1414.59 ± 246.21 ab	175.80 ± 53.76 b	5.59 ± 0.18 a	3.01 ± 0.84 abc
CK-7	0	7	1749 ± 413 a	107 ± 13 bc	1825.86 ± 443.70 a	122.28 ± 12.89 bcd	5.70 ± 0.34 a	3.10 ± 0.38 abc
L-7	0.5	7	1438 ± 242 ab	104 ± 22 bc	1490.16 ± 239.13 ab	115.49 ± 34.19 bcd	5.75 ± 0.20 a	2.60 ± 0.67 abc
M-7	1.5	7	1326 ± 513 ab	132 ± 18 bc	1358.64 ± 540.61 ab	156.43 ± 36.37 bcd	5.22 ± 1.04 a	2.83 ± 0.64 abc
H-7	3.0	7	1499 ± 362 ab	136 ± 38 bc	1554.74 ± 390.70 ab	164.57 ± 49.86 bc	5.55 ± 0.48 a	2.24 ± 0.91 bc
CK-14	0	14	1363 ± 121 ab	80 ± 11 c	1401.94 ± 116.97 ab	84.71 ± 11.54 cd	5.79 ± 0.11 a	2.39 ± 0.37 abc
L-14	0.5	14	1027 ± 200 b	128 ± 14 bc	1048.92 ± 216.46 b	146.10 ± 19.68 bcd	4.82 ± 0.72 a	3.11 ± 1.22 abc
M-14	1.5	14	1332 ± 114 ab	70 ± 20 c	1369.43 ± 121.93 ab	76.80 ± 15.63 d	5.48 ± 0.30 a	2.01 ± 1.25 c
H-14	3.0	14	1364 ± 389 ab	228 ± 82 a	1409.17 ± 402.55 ab	258.08 ± 84.25 a	5.59 ± 0.30 a	3.60 ± 0.24 a

Each value is the mean of three replicates ± SD. Means with different lowercase letters indicate significant differences between samples at *p* < 0.05 level according to Dunn’s post hoc test. “-” means adding nothing.

## Data Availability

Not applicable.

## Supplementary Materials

The following supporting information can be downloaded at: https://www.mdpi.com/article/10.3390/plants11243550/s1, Table S1: Chemical composition of the volatile oils from *Amomum villosum* Lour.
