# Supplementary material for: Allelopathic Effects of Amomum villosum Lour. Volatiles from Different Organs on Selected Plant Species and Soil Microbiota
Source: Plants (Basel). 2022 Dec 16;11(24):3550. doi: 10.3390/plants11243550 (PMC9781342; doi:10.3390/plants11243550)
Supplement: Supplementary file 1 [file plants-11-03550-s001.zip › plants-2043992-supplementary.pdf]

Supplementary Table S1. Chemical composition of the volatile oils from *Amomum villosum* Lour.

Number	Compounds	Percentage (%)			
		Stem	Leaf	Young fruit	Whole plant
1	E-2-Hexen-1-al	-	0.67	-	-
2	3-Hexen-1-ol	-	-	-	0.38
3	Tricyclene	-	-	0.20	-
4	Thujene	0.61	0.34	0.13	0.57
5	α-Pinene	**16.26**	**28.18**	4.30	**15.03**
6	Camphene	-	0.28	8.83	0.87
7	β-Phellandrene	**24.65**	8.26	-	**15.14**
8	Sabinene	-	-	1.50	-
9	β-Pinene	**28.30**	**48.62**	4.84	**23.70**
10	β-Myrcene	1.46	0.73	2.81	1.49
11	α-Phellandrene	-	-	0.38	0.08
12	Terpinene	1.35	0.34	0.52	1.00
13	*p*-Cymene	1.04	0.14	0.06	0.42
14	D-Limonene	2.15	2.16	7.75	3.75
15	Eucalyptol	0.27	-	0.12	0.12
16	Ocimene	-	0.07	0.02	0.09
17	γ-Terpinene	2.46	0.66	0.81	1.93
18	β-Terpineol	0.25	-	0.02	0.08
19	Terpinolene	0.72	0.22	0.40	0.59
20	Linalool	0.19	-	1.93	0.32
21	Fenchyl alcohol	-	-	0.02	0.03
22	2-Cyclohexen-1-ol, 1-methyl-4-(1-methylethyl)-, *trans-*	0.32	-	0.07	0.19
23	α-campholenal	-	-	-	0.05
24	L-Pinocarveol	-	0.18	-	0.45
25	2-Cyclohexen-1-ol, 1-methyl-4-(1-methylethyl)-, *cis-*	-	-	0.06	-
26	Camphor	-	0.33	**16.58**	2.65
27	Camphenehydrate	-	-	0.15	-
28	Isoborneol	-	-	0.27	-
29	Pinocarvone	-	-	-	0.32
30	Borneol	-	0.09	4.51	0.36
31	Terpinen-4-ol	6.32	1.38	1.65	4.07
32	α-Terpineol	0.72	0.32	0.44	0.64
33	Myrtenal	-	0.37	0.03	0.87
34	trans-Piperitol	-	-	0.03	0.11
35	Verbenone	-	-	-	0.04
36	Carveol	-	-	-	0.03
37	Fenchyl acetate	-	-	0.05	-
38	Bornyl formate	-	-	0.06	-
39	Carvone	-	-	-	0.03

40	Bornyl acetate	0.46	1.35	**38.30**	4.03
41	Myrtenyl acetate	-	-	-	0.05
42	*p*-Mentha-1,4-dien-7-ol	-	-	-	0.05
43	Copaene	-	-	0.15	0.11
44	β-Elemene	1.00	0.08	0.10	0.59
45	α-Santalene	-	-	0.04	0.04
46	Caryophyllene	1.15	0.42	0.16	0.98
47	α-Ionone	-	-	-	0.06
48	α-Bergamotene	-	-	0.04	0.06
49	β-Farnesene	-	-	0.04	0.05
50	1,4,7,-Cycloundecatriene, 1,5,9,9-tetramethyl-, Z,Z,Z-	0.20	0.06	0.04	0.21
51	Aromadendrene	-	-	0.03	0.17
52	Naphthalene,1,2,3,5,6,7,8,8a-octahydro-1-methyl-6-methylene-4-(1-methylethyl)-	-	-	0.02	-
53	γ-Elemene	1.94	0.41	0.29	1.54
54	Cyclohexene, 1-methyl-4-(5-methyl-1-methylene-4-hexenyl)-, (S)-	-	-	0.04	-
55	8-Isopropenyl-1,5-dimethyl-cyclodeca-1,5-diene	-	-	0.06	0.22
56	Cadinene	-	-	0.15	0.12
57	(-)-Spathulenol	0.46	-	0.02	0.28
58	Caryophyllene oxide	0.40	0.09	0.08	0.32
59	Viridiflorol	-	-	0.05	0.08
60	Naphthalene, 1,2,3,4,4a,7-hexahydro-1,6-dimethyl-4-(1-methylethyl)-	-	-	0.04	0.02
61	T-Muurolol	-	-	0.07	-
62	α-Cadinol	-	-	0.10	-
63	Ar-turmerone	-	-	0.02	-
64	Santalol	-	-	0.12	-
65	Bergamotenol	-	-	0.10	-
66	Farnesyl acetone	-	-	-	0.14
67	Eicosane	-	-	0.03	-
	Monoterpene hydrocarbons	79.00	89.99	32.55	64.66
	Oxygenated monoterpenes	8.53	4.02	64.29	14.49
	Sesquiterpene hydrocarbons	4.29	0.97	1.18	4.11
	Oxygenated sesquiterpenes	0.86	0.09	0.57	0.88
	Others	-	0.67	0.03	0.38
	Total identified	92.67	95.74	98.62	84.52
	Oil yield (%, v/w)	0.15	0.40	0.50	0.20

"-" represent not detected. Bold numbers represent the percentage of chemicals more than 15% in the organ.
